# Rechallenge and maintenance therapy using cetuximab and chemotherapy administered to a patient with metastatic colorectal cancer

**DOI:** 10.1186/s12885-017-3133-8

**Published:** 2017-02-14

**Authors:** Jian Ma, Quan-liang Yang, Yang Ling

**Affiliations:** Changzhou Cancer Hospital of Soochow University, Changzhou, Jiangsu Province 213032 People’s Republic of China

**Keywords:** Cetuximab, Metastatic colorectal cancer, Rechallenge, Maintenance therapy

## Abstract

**Background:**

Cetuximab combined with chemotherapy is one of the first-line treatments of metastatic colorectal cancer. Although disease progression inevitably occurs, rechallenge and maintenance therapies using cetuximab-based regimens may be beneficial, particularly for patients with wild-type (WT) *KRAS*.

**Case presentation:**

A 47-year-old female patient who underwent right hemicolectomy presented with an ulcerative adenocarcinoma (grade 2) revealed by histopathological analysis. The patient received three cycles of adjuvant chemotherapy, but disease recurred 15 months later. Cetuximab and a FOLFOX-4 regimen were administered, followed by surgery and adjuvant chemotherapy that was administered for approximately one year. Three years after completing adjuvant therapy, her serum carcinoembryonic antigen levels rapidly increased, and enhanced computed tomography showed widespread metastases. Rechallenge with cetuximab and the FOLFIRI regimen was then initiated, and after 12 cycles, lesions in the lung and liver shrank significantly, and serum CEA levels dramatically declined. Maintenance therapy with cetuximab and capecitabine was then administered for 10 months until the metastatic lesions in the lung and liver enlarged.

**Conclusion:**

Rechallenge and maintenance therapy with cetuximab-based chemotherapy were relatively effective for managing a female patient with WT *KRAS*. Optimization of this strategy requires further in-depth investigations of more patients.

## Background

Colorectal cancer (CRC) is one of the most common cancers worldwide. Approximately 60% of patients are diagnosed at a relatively late stage, and 71% with regionally distributed disease survive for 5 years. However, when disease has spread to distant organs, 5-year survival decreases to 13% [1]. One of the first-line options for treating patients with advanced CRC is the monoclonal antibody cetuximab that targets the epidermal growth factor receptor (EGFR) [2–4]. When combined with chemotherapy, cetuximab significantly improves progression-free survival and median overall survival of patients with wild-type (WT) KRAS [2, 3]. However, acquired resistance caused by a secondary mutation of *KRAS* occurs at a relatively high rate during cetuximab treatment [5]. For patients with WT *KRAS*, rechallenging initial responders with cetuximab-based regimens represents a promising strategy [6–8]. Here we present the case of a female patient who received three lines of therapy following her second surgery. Unfortunately, the disease metastasized to numerous organs, accompanied by a dramatic increase in serum carcinoembryonic antigen (CEA) levels before the rechallenge regimen commenced. The rechallenge and the subsequent maintenance therapies were highly beneficial for reducing the size of the lesions in lung and liver and reduced CEA levels.

## Methods

The drugs and antibodies used are as follows:

Cetuximab (Erbitux): Merck Serono Co., Ltd.

Capecitabine (Xeloda): Shanghai Roche Pharmaceuticals Ltd.

Bevacizumab (Avastin): Shanghai Roche Pharmaceuticals Ltd.

Oxaliplatin and irinotecan: Jiangsu Hengrui Medicine Co., Ltd

## Case presentation

A 47-year-old woman who had undergone resection of the ascending colon on November 16, 2006, was admitted to our Department of Oncology with a diagnosis after surgery of ulcerative adenocarcinoma (grade 2) of the ascending colon. The tumor penetrated to the surface of the visceral peritoneum, and metastases were detected in five regional lymph nodes (pT4aN2aM0). In accordance with National Comprehensive Cancer Network Guidelines in Oncology [9], we initiated adjuvant therapy using the XELOX regimen comprising oxaliplatin (130 mg/m^2^, Day 1) and capecitabine (1000 mg/m^2^, p.o bid Days 1–14), 21 days per cycle. After three cycles, the patient refused to continue therapy due to the onset of leukopenia (grade 3), in accordance with the guidelines of the NCI Common Terminology Criteria for Adverse Events (CTCAE v 4.0) [10].

On February 20, 2008, a routine examination revealed that her serum carcinoembryonic antigen (CEA) concentration increased to 24.41 ng/ml without symptoms. The results of positron-emission tomography with computed tomography (PET-CT) showed increased metabolic activities of the right adnexa mass and hepatic hilar region node. At that time, her *KRAS* status was unknown and such testing was not a mandatory requirement of the China Food and Drug Administration (CFDA). On March 1, 2008, the patient started to receive cetuximab and the FOLFOX4 regimen (Day 1: 5-FU 400 mg/m^2^ bolus injection; LV 200 mg/m^2^; 22 h continuous infusion with 5-FU 600 mg/m^2^, and oxaliplatin 85 mg/m^2^; Day 2: the same regimen without oxaliplatin, each 14-day cycle). Cetuximab was administered (initial dose of 400 mg/m^2^) followed by 250 mg/m^2^ per week. Four cycles later, abdominal enhanced CT demonstrated a partial response (PR) of the metastases according to the Response Evaluation Criteria in Solid Tumors (RECIST 1.0), and the patient underwent right adnexectomy and partial gastrointestinal ligament resection. One month after surgery, two additional cycles of FOLFOX4 regimen (see above) were administered. However, we changed to the FOLFIRI regimen for two cycles because of neuropathy (sensory, grade 2) caused by oxaliplatin and the patient’s refusal of FOLFOX4. However, because the patient suffered from the severe chest tightness and fatigue (grade 2), we discontinued the FOLFIRI regimen and started oral administration of capecitabine (1000 mg/m^2^, p.o bid, Days 1–14 every three weeks for 10 months).

**Fig. 1:**
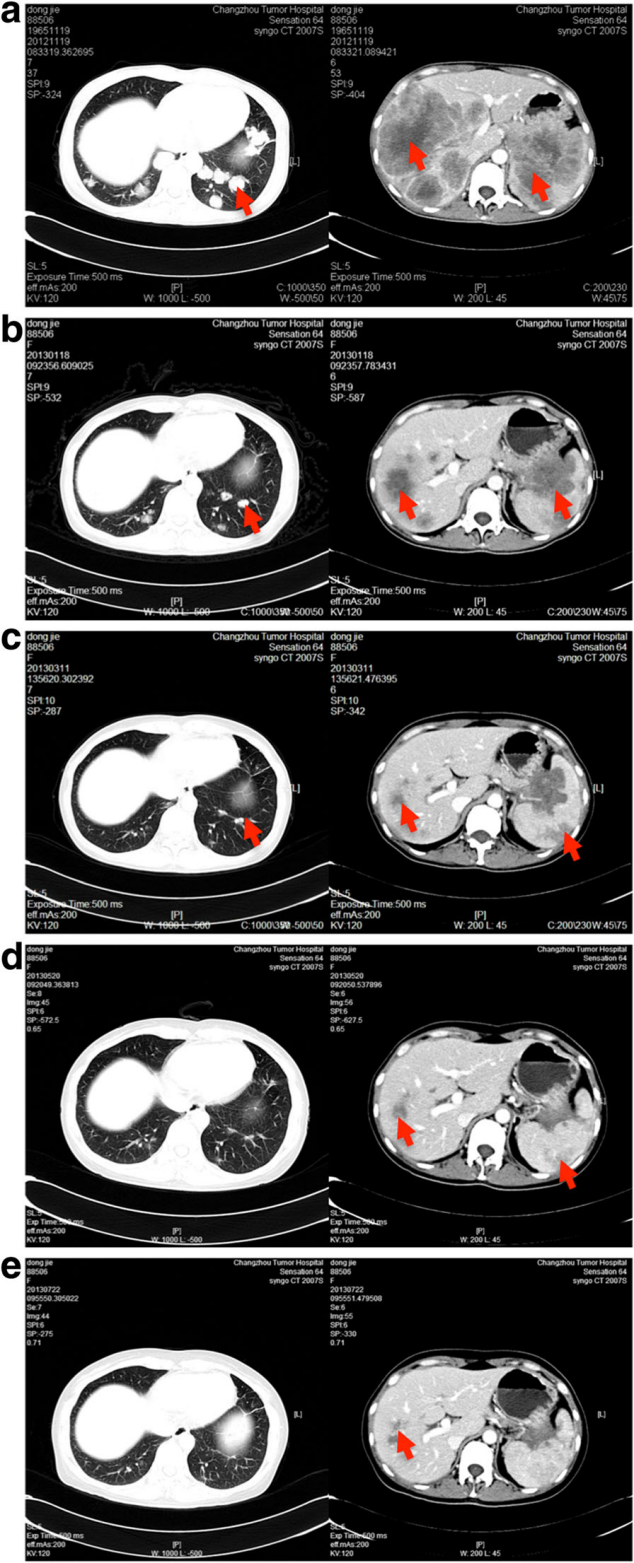
CT results showing the metastatic lesions in the liver, lung and kidney before (**a**), four (**b**), eight (**c**), and twelve cycles (**d**) after cetuximab and FOLFIRI rechallenge, and two cycles (**e**) after cetuximab and capecitabine maintenance therapy. Arrows indicate the lesions

**Fig. 2:**
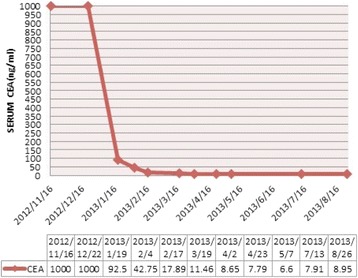
CEA levels of the patient

During the next three years, routine examinations did not detect recurrence, although the patient experienced pain around the anus. In November 2012, her serum CEA increased to >1,000 ng/ml. CT of the chest and abdomen revealed widely distributed metastases, including multiple involved lymph nodes in the mediastinum, pelvic cavity, and behind the peritoneum, liver, and both lungs (Fig. 1a). Molecular analysis identified WT *KRAS*. From November 2012, the patient was rechallenged weekly with cetuximab (400 mg/m^2^ initially and 250 mg/m^2^ thereafter) and FOLFIRI (Day 1: Irinotecan 180 mg/m^2^; 5-FU 400 mg/m^2^ bolus injection; 5-FU 600 mg/m^2^, 22 h continuous infusion; LV 200 mg/m^2^; Day 2:the same regimen without irinotecan; each 14-day cycle). After two cycles of treatment, the pain around the anus was relieved, although the CEA concentration did not decline (>1000 ng/ml) (Fig. 2). Four cycles later, chest and abdominal enhanced CT demonstrated that all the lesions shrank significantly (PR, according to RECIST 1.1) (Fig. 1b), and the CEA concentration was significantly reduced to 92.5 ng/ml (Fig. 2). Eight and twelve cycles later, chest and abdominal enhanced CT showed that all lesions shrank further and the CEA concentrations were reduced to 17.89 ng/ml and 6.6 ng/ml, respectively (Figs. 1c, d and Fig 2). During treatment, rash, eyelash trichomegaly and myelosuppression (grade 2) occurred.

We then changed the treatment strategy to capecitabine single-agent chemotherapy (750 mg/m^2^ p.o bid, Days 1–21, each 28-day cycle) combined with cetuximab (250 mg/m^2^ weekly) as maintenance therapy, which led to further shrinkage of the lesions after two cycles (Fig. 1e). We continued maintenance therapy for approximately 10 months (April 2014), when the lesions in the lung and the liver progressed (PD). Peripheral neuropathy was improved at that time, and the patient had a strong desire to survive; she therefore accepted our advice and tried the FOLFOX4 regimen again, risking its potentially severe side effects. We switched to bevacizumab (5 mg/kg, once every two weeks) plus the FOLFOX4 regimen for four cycles. CT showed stable disease; however, she refused intravenous chemotherapy because of intolerance. We therefore replaced FOLFOX4 with capecitabine. Progression-free survival lasted from April through November, and she was then administered regorafenib for nearly two months, which was terminated because of financial difficulties. We then administered cetuximab plus capecitabine for approximately three months, followed by the best available supportive care until she died on August 29, 2015.

## Discussion

The cetuximab-based regimen is one of the first-line options for treating patients with advanced colorectal cancer. Cetuximab is a chimeric IgG1 monoclonal antibody against EGFR that inhibits the ligand-induced EGFR signaling pathway through binding to the extracellular domain of EGFR, which inactivates EGFR and its downstream factors [11].

Clinical trials demonstrate the benefit of cetuximab in combination with chemotherapy for treating patients with metastatic colorectal cancer (mCRC) [12, 13]. However, because mCRC is heterogeneous, cetuximab-based regimens only benefit patients with WT *KRAS*, a downstream effector of the EGFR signaling pathway [14]. Mutational activation of *KRAS* can confer primary resistance to EGFR-targeted therapies, including cetuximab [12–14]. Nevertheless, for initial responders who harbor WT *KRAS* or silent *KRAS* mutations, rechallenge with cetuximab may be further clinically beneficial if patients do not respond to a new line chemotherapy and therefore receive other therapies [8].

Several clinical trials tested this hypothesis and demonstrated favorable efficacies of cetuximab-based rechallenge regimens [8, 15]. Notably, rebiopsy may be required when rechallenge is considered for these patients, because secondary *KRAS* mutations may confer acquired resistance to EGFR-targeted therapy [5]. Here we report a female patient who was administered three lines of therapy after the second surgery without an activating *KRAS* mutation. Rechallenge with cetuximab caused a beneficial and dramatic shrinkage of metastatic lesions in the lung and liver as well as a significant reduction in CEA levels, further supporting the expectation of a promising outcome of a rechallenge using cetuximab.

Maintenance therapy is an important approach for improving the outcomes of patients with cancer who receive certain lines of chemotherapy to prolong the duration of therapy to control long-term cancer growth. The phase III CAIRO3 trial explored the efficacy of maintenance therapy with capecitabine plus bevacizumab, compared with the observation group in patients who achieve at least stable disease after six cycles (18 weeks) of induction therapy with capecitabine, oxaliplatin and bevacizumab (CAPOX-B) [16]. The conclusion drawn from this trial was that maintenance therapy significantly delayed tumor progression and did not compromise a patient’s quality of life. However, the effect of cetuximab-based maintenance therapy has not been conclusively investigated.

According to the findings of the CAIRO3 trial, we tested here the efficacy of maintenance therapy using cetuximab and capecitabine and found further shrinkage of metastatic lesions after two cycles. The disease progressed after 10 months of maintenance therapy, suggesting that in addition to rechallenge, maintenance treatment with a cetuximab-based regimen may potentially benefit the patient. Further validation and optimization of this strategy with more patients should be conducted.

Optimizing the sequence of administration of cetuximab and bevacizumab may influence overall survival, because the CRYSTALY and FIRE-3 trials found that early tumor shrinkage was more likely to occur after cetuximab treatment that improves the R0 removal rate of the tumor [4, 12]. Further, single-agent maintenance therapy using cetuximab should be evaluated to determine the efficacy of cetuximab in patients treated with FOLFIRI, as reported in the MACRO-II trial, which found that cetuximab alone achieves similar benefits with fewer side-effects compared with mFOLFOX plus cetuximab [17]. Moreover, it will likely be informative to evaluate the tumor response to reintroduction of FOLFIRI plus cetuximab when maintenance therapy fails, as indicated by the reintroduction of CAPOX-B in the CAIRO-3 trial [16].

A recent complete exome sequencing study identified mutations in ERBB2, EGFR, FGFR1, PDGFRA and MAP2K1 as potential drivers of resistance to EGFR-targeted therapies [18]. Therefore, it may be useful to determine the efficacies of approaches that target these genes in combination with cetuximab for rechallenge and maintenance treatment.

The patient died more than one year before the preparation of this manuscript. The subsequent emergence of new treatments has provided more options for patients with similar characteristics to the present patient. For example, trifluridine-tipiracil (TAS-102) is approved by the FDA, and in the treatment of refractory colorectal cancer, the median overall survival improved from 5.3 months with placebo to 7.1 months with TAS-102 [19]. Pembrolizumab is active in patients with mismatch repair-deficient colorectal cancer (response rate, 40%; 95% confidence interval, 12 – 74%) [20]. These regimens may achieve better outcomes.

## Conclusion

Rechallenge with cetuximab and FOLFIRI may be effective for treating patients with mCRC, and subsequent maintenance therapy with cetuximab and capecitabine may provide an option for selected patients.

## Abbreviations

CEA: Carcinoembryonic antigen
CFDA: China Food and drug administration
CT: Computed tomography
EGFR: Epidermal growth factor receptor
FOLFIRI: Folinic acid, fluorouracil and irinotecan
mCRC: Metastatic colorectal cancer
PET-CT: Positron-emission tomography with computed tomography
WT: Wild-type
